# ITGA6 gene silencing by RNA interference modulates the expression of a large number of cell migration-related genes in human thymic epithelial cells

**DOI:** 10.1186/1471-2164-14-S6-S3

**Published:** 2013-10-25

**Authors:** Daiane Cristina Ferreira Golbert, Eliane Correa-de-Santana, Marcelo Ribeiro-Alves, Ana Tereza Ribeiro de Vasconcelos, Wilson Savino

**Affiliations:** 1 Laboratory on Thymus Research, Oswaldo Cruz Institute, Oswaldo Cruz Foundation, Rio de Janeiro, Brazil; 2 Evandro Chagas Research Institute, Oswaldo Cruz Foundation, Rio de Janeiro, Brazil; 3 Bioinformatics Laboratory, National Laboratory of Scientific Computation, Petrópolis, Brazil

## Abstract

**Background:**

The thymic epithelium is the major microenvironmental component of the thymus, the primary lymphoid organ responsible for the generation of T lymphocytes. Thymic epithelial cells (TEC) control intrathymic T cell differentiation by means of distinct types of interactions. TEC constitutively produce chemokines and extracellular matrix ligands (such as laminin and fibronectin) and express corresponding receptors, which allow thymocytes to migrate in a very ordered fashion. We previously showed that laminin mediates TEC/thymocyte interactions in both mice and humans. More recently, we used RNAi technology to knock-down the ITGA5 gene (which encodes CD49e, the integrin α-chain subunit of the fibronectin receptor VLA-5) in cultured human TEC. Using a similar strategy, herein we knocked-down the ITGA6 gene, which encodes CD49f, the α-chain of two integrin-type laminin receptors, namely VLA-6 (α6β1) and α6β4.

**Results:**

We first confirmed that RNAi-induced knock-down of the ITGA6 gene was successful, at both transcription and translational levels, with a significant decrease in the membrane expression of CD49f, apart from CD49b, CD49c and CD49d, ascertained by cytofluorometry on living TEC. We also demonstrated that such knock-down promotes a decrease in cell adhesion to laminin. Using quantitative PCR, we demonstrated that gene expression of other integrin α-chains were concomitantly down-regulated, particularly those which form other laminin receptors, including ITGA1, ITGA2 and ITGA7. Interestingly enough, LAMA1 gene expression (whose corresponding protein chain is part of laminin-111) was largely increased in ITGA6 knocked-down TEC cultures. Lastly, the network complexity of gene expression under ITGA6 influence is much broader, since we found that other cell migration-related genes, namely those coding for various chemokines, are also modulated when IGTA6 is knocked-down.

**Conclusion:**

The data presented herein clearly show that down regulation of ITGA6 gene in the human thymic epithelium triggers a complex cascade of effects upon the expression levels of several other cell migration-related genes, including extracellular matrix and chemokine ligands and receptors. Taken together, these data unravel the concept that the expression of genes involved in controlling of thymocyte migration by the thymic microenvironment should be regarded as complex networks, so that a defect in the expression of one single gene may reflect in an amplified cascade with functional consequences for TEC adhesion onto the natural ligand and potential consequences upon the normal patterns of TEC/thymocyte interactions.

## Background

The thymus is a central lymphoid organ, in which bone marrow-derived T cell precursors undergo a complex process of maturation, eventually leading to the migration of positively selected thymocytes to the T-dependent areas of peripheral lymphoid organs. It has been largely demonstrated that, for such a process to occur normally, it is crucial that developing thymocytes interact to non-lymphoid cells of the organ, the thymic microenvironment [1]. This tridimensional network is composed of a variety of cell types; the thymic epithelial cells (TEC) being the most conspicuous elements, although nonepithelial dendritic cells, macrophages and, to a lesser extent fibroblasts, also play a role in the general process of intrathymic T cell differentiation. In this context, a group of interactions between developing thymocytes and microenvironmental cells is mediated by extracellular matrix (ECM) ligands and receptors [2,3], and there is evidence that ECM molecules play a relevant role in localizing the different thymocyte stages of differentiation in discrete niches within the thymic lobules [4]. Moreover, supramolecular ECM arrangements may function as a conveyor belt, allowing an ordered migration of thymocytes within the organ [5]. In this context, we have demonstrated that interactions mediated by fibronectin and its receptor VLA-5 (the integrin α5β1 or CD49e/CD29) influence TEC thymocyte adhesion, as demonstrated by the use of neutralizing anti-CD49e monoclonal antibodies [6] or RNA interference to abrogate translation of the CD49e protein [7]. Such functional relevance is not restricted to interactions mediated by fibronectin, but is rather extended to other ECM ligands. We demonstrated in both mice and humans that developing thymocytes and TEC express constitutively the laminin receptor VLA-6 (the integrin α6β1 or CD49f/CD29), which also participates in heterocellular cell adhesion and migration events, which could be significantly disrupted by using anti-VLA-6 antibody applied to growing TEC prior to co-culturing with thymocytes [8-10].

In addition to VLA-6, another integrin-type laminin receptor, VLA-3 (α3β1 or CD49c/CD29), was suggested to play a role in thymocyte adhesion to laminin [11]. Moreover, we investigated the expression and *in situ *localization of laminin chains in the human thymic tissue, as well as in the lymphoepithelial complex named thymic nurse cells. Also, we showed that TEC produce laminin and grows faster in the presence of this molecule in culture dishes [12,13].

More recently, we showed that TEC control thymocyte migration in a multivectorial way, with each ligand/receptor pair contributing to one given vector [14]. Furthermore, we found that one given interaction can modulate another interaction [15,16], thus placing the multivectorial concept for intrathymic T cell migration still more complex.

In order to better understand the role of laminin receptors in the behavior of thymic epithelial cells we used herein RNA interference to impair the expression of CD49f (thus preventing the normal expression of two laminin receptors α6β1 and α6β4) in cultured human TEC. This strategy resulted in the down modulation, not only of CD49f, but also other integrin α-chains of both laminin and fibronectin receptors. Most importantly, we found that several other cell migration-related genes were modulated secondary to treating human TEC with ITGA6 specific siRNA, thus pointing to the notion that intra-TEC expression of genes involved in controlling thymocyte migration by the thymic microenvironment should be regarded as complex networks. These gene modulations parallel the decrease in TEC ability to adhere onto a laminin coat and to assume the normal stellate shape.

## Results

### Knock-down of ITGA6 in human thymic epithelial cells promotes adhesion defects

**Figure 1:**
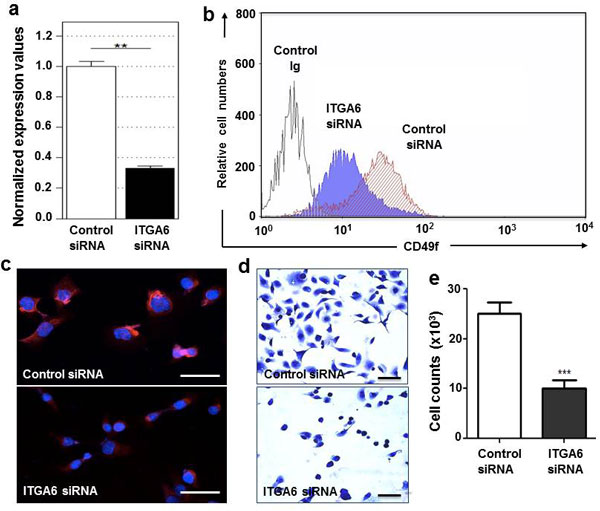
**Knock-down of ITGA6 affect adhesion of human thymic epithelial cells**. TEC were transfected either with siRNA oligonucleotides targeting the ITGA6 gene (ITGA6 siRNA) or with a scramble siRNA oligonucleotide sequence (Control siRNA). Results of TEC treated either by using control siRNA or ITGA6 siRNA are presented in each assay. ITGA6 gene expression was measured in five independent transfection experiments by qRT-PCR, seen in panel **a**. The protein encoded by ITGA6 gene, CD49f, was evaluated on the surface of TEC by cytofluorometry after gene silencing experiments (panel **b**). Gray histogram represents control isotype, the filled histogram depicts the cells transfected with ITGA6 siRNA and the hachured are the cells with control siRNA. CD49f expression was also determined by immunofluorescence, as shown in panel **c**. In all cases, gene expression and the corresponding integrin α-chain were consistently and significantly impaired. Transfected TEC were plated onto laminin coat (d). Note that the control cells (control siRNA) attached and spread well than knocked cells (ITGA6 siRNA). The cell number in both condition were quantified (e). The individual bars represent mean ± standard error (n = 5). Magnification bars correspond to 35 µm in panel **c **and 70 µm in panel **d**. All experiments were done 48 hours of gene silencing and using a siRNA dose of 5nM. **p < 0.01.

We successfully silenced ITGA6 gene in cultured human thymic epithelial cells by siRNA, using specific anti-ITGA6 oligonucleotide. As compared to scramble oligonucleotide, ITGA6 RNA message in TEC transfected with ITGA6 siRNA was reduced 70-80%, as seen by qPCR (Figure 1a). Such reduction could be clearly seen 48 hours after transfection. Importantly, this effect was followed by a significant inhibition of the CD49f protein, as ascertained by cytofluorometry and immunofluorescence (Figure 1b-c). Worth of note, untreated TEC compared with scramble siRNA treated counterparts exhibited similar CD49f membrane expression levels (not shown).

Next, we examined whether ITGA6 knocked-down TEC showed any defects in their response to laminin. Actually, α6 integrin-knocked down TEC showed marked adhesive defects to laminin with impairment as compared to control siRNA-treated cells. Quantitation of this adhesion loss revealed a 60% decrease in cell adhesion to laminin, as compared to control. Moreover, ITGA6 knocked-down TElose their typical stellate profile in acquire the cell shape (Figures 1d-e).

Since many of the ITGA6 siRNA-treated TEC exhibited a smaller size, compared to controls, we could argue that significant amounts of these TEC actually corresponded to dying cells. We approached this issue by flow cytometry, analyzing forward (FSC) and side scatter (SSC) profiles. This strategy allows us to indentify dying cells, by their very low FCS. Nevertheless, in five independent experiments the cytofluorometric profiles in both groups were essentially the same in terms of the relative numbers of very low FSC pattern (see Additional file 1).

### siRNA-mediated ITGA6 gene silencing in human TEC promotes a decrease in the expression levels of other integrin-type ECM receptors

**Figure 2:**
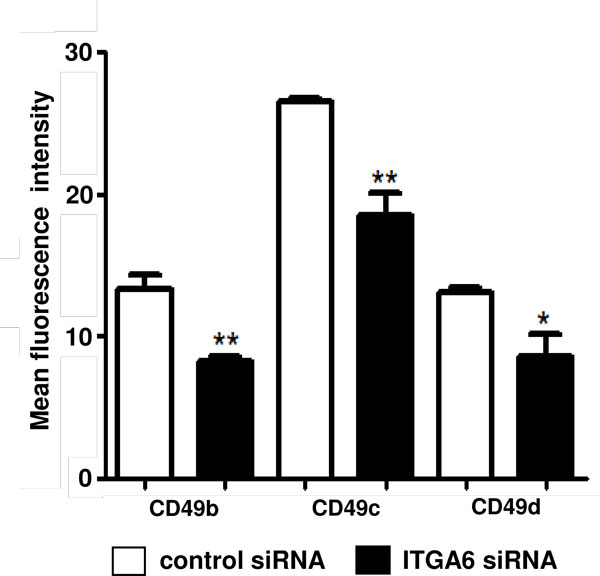
**ITGA6 gene silencing in human TEC down-regulates the membrane expression of other integrin α-subunits**. Membrane expression of the integrin α-chains CD49b, CD49c and CD49d was evaluated by cytofluorometry and data are expressed as mean fluorescence intensity. The individual bars on the graph represent mean ± standard error (n = 4), and *p *-values were generated using Student's *t*-test. *p < 0.05 and **p < 0.01.

We next investigated whether the surface expression of other integrin-type ECM receptor could be altered in ITGA6-siRNA treated human TEC. For that, we evaluated by flow cytometry of unfixed cells the membrane density of CD49b, CD49c and CD49d, the integrin α-chains for the receptors α2β1 (VLA-2), α3β1 (VLA-3) and α4β1 (VLA-4). In all cases, there was a significant decrease in the mean fluorescence intensity in ITGA6 siRNA treated TEC, when compared cultured TEC incubated with the scramble siRNA (Figure 2). Differently, membrane density of CD29 (the β1 integrin chain) was similar in both groups (not shown), suggesting that not all β1-containing integrins expressed by human TEC are down regulated concomitantly.

### Multiple cell migration-related genes are modulated in human TEC after ITGA6 gene silencing

**Figure 3:**
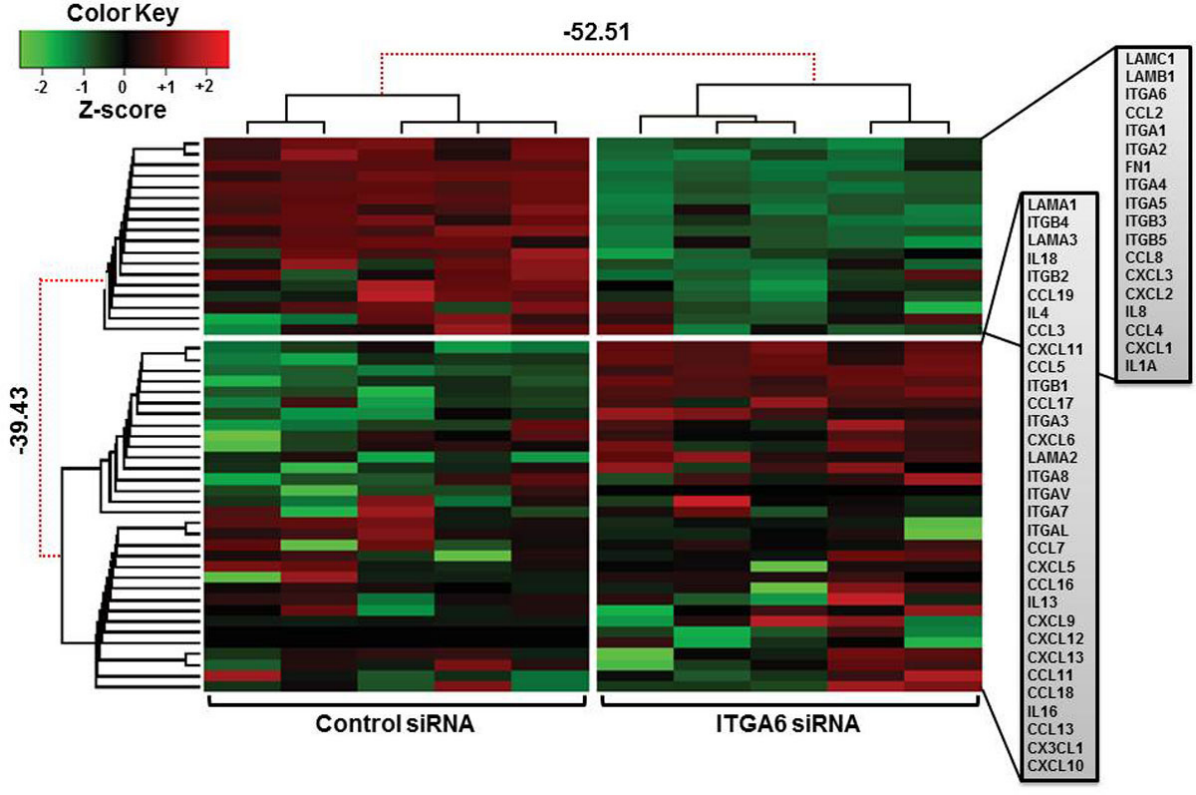
**Differential expression of multiple cell migration-related genes in human thymic epithelial cells after ITGA6 gene knock-down**. The figure shows heat map of gene expression for selected extracellular matrix and chemokine ligand and receptor genes. Differential expression was defined with PCR arrays, comparing ITGA6 siRNA treated TEC with control siRNA. Bayesian gene clustering was done with sample profiles in ITGA6 siRNA silenced TEC (n = 5) *versus *control siRNA-treated TEC (n = 5). The graphic displays dendrograms representing the 1D clusterization of genes (left) and samples (top) and the 2D map corresponding to the levels of standardized gene expression profiles (z-score). The green areas display a decreased expression of the analyzed transcripts of the ITGA6 siRNA group, in comparison to the expression in the control siRNA group (down-regulated) and the red areas display an increased expression (up-regulated). Red dotted lines in dendrograms (left-left and top-left) indicate statistic significant weak unions, discouraged by the Bayesian clustering analysis. Values represented in the dendrogram branches correspond to log-odds of the union of corresponding branches. Colorkey indicate gene z-scores on "Control siRNA" and ITGA6 siRNA samples. The clustered genes are listed in gray rectangles on the right side of the figure.

The above results prompted us to design experiments aiming to search for putative modulation of a large series of cell migration-related genes, comprising not only integrins but also laminins, as well as chemokines, known to be produced by TEC and to play a role in interactions with developmental thymocytes [2,4]. Overall, we evaluated by quantitative RT-PCR 50 genes (see Table 1, Additional file 2) and compared their expression levels in ITGA6 siRNA *versus *control siRNA treated TEC. Figure 3 shows the heat map and dendrograms representing the bi-cluster analysis of these experiments (n = 5). Statistical comparison allowed us to define that the gene expression patterns differed between the two groups, with 28 genes (out of the 50 assayed) being either down- or up-regulated. Among the clear-cut down-regulated cluster we found 18 genes, several of them encoding integrin and laminin chains.

Particularly, the expression of ITGA7 (encoding for the α-7 integrin chain of the α7β1 laminin receptor) and ITGA6 (encoding for the α-6 integrin chain of the α6β1 laminin/collagen receptor) were significantly decreased. Gene regulation induced by ITGA6 knock-down in the human thymic epithelium is far more complex, since 10 genes were significantly up-regulated in ITGA6 siRNA-treated TEC as compared to controls, and also included genes encoding other integrin and laminin chains. Interestingly enough, LAMA1 gene expression (whose corresponding protein chain is part of laminin-111) is largely increased in ITGA6 knocked-down TEC cultures suggesting a compensatory upregulation of this gene, whereas LAMB and LAMC genes are down-regulated. One could argue that these gene modulations were off-target effects. However, this does not seem to be the case, since when we applied a BLAST analysis with the six siRNA sequences (sense and anti-sense). We have not find a single target in the human transcriptome with e-value ≤ 0.006, thus strongly indicating the specificity of siRNA to the ITGA6 mRNA (see Table 2, Additional file 3). Lastly, the network complexity of gene expression under ITGA6 influence in human TEC is much broader, since we found that other cell migration-related genes, namely those coding for a variety chemokines, were also modulated when IGTA6 was knocked-down. Among them, six chemokines genes were down-regulated and three were up-regulated, whereas the expression of 14 genes did not differ significantly. Furthermore, among six cytokine genes included in the analysis, one was down-regulated (IL-8) and two were up-regulated (IL-4 and IL-18).

### Potential networks of cell migration-related gene interactions that can be disrupted in human TEC after ITGA6 gene silencing

**Figure 4:**
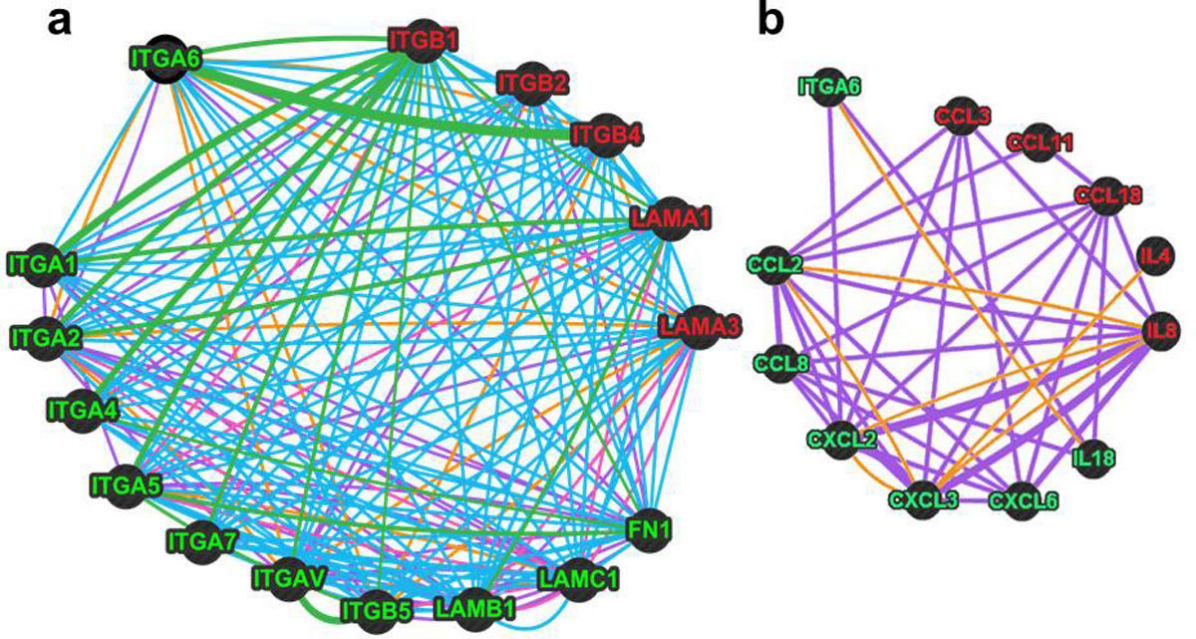
**Cell migration-related networks potentially affected by knocking-down the ITGA6 gene in the human thymic epithelium**. Panel **a **depicts the ITGA6 gene and the selected experimental down- or up-regulated gene expression (green and red spheres, respectively), corresponding to integrin as well as laminin chains. Panel **b **shows the network of interactions involving ITGA6 and chemokines genes that have been experimentally down- or up-regulated after ITGA6 knock-down. Networks of potential interactions were drawn within the framework of the *GeneMANIA database *(http://www.genemania.org). Accordingly, each line represents a given type of functional association, available in the literature derived from various biological systems and adopted by *GeneMANIA *algorithm. Green lines = physical interactions; violet lines = co-expression; orange lines = co-localization; blue lines = metabolic pathways; pink lines = genetic interactions. The thicker is each line, the closer is the weight of association of the two given genes to each other. Overall panel **a **indicates that the up- or down genes, experimentally defined may them cross-interact, thus impacting the cascade of changes induced by this single gene knock-down in the human thymic epithelium. Additionally, panel **b **suggests that most of the changes seen in chemokine gene modulation occur via the changes directly induced upon the expression of CXCL2 and IL18.

Considering that the experimental results described above point out to a complexity of gene regulation in ITGA siRNA treated human TEC, we evaluated *in silico *potential interaction networks that may be affected by ITGA6 knock-down. For that, we applied the bioinformatics tools available at *GeneMANIA *(see material & methods session). We inserted separately ITGA6 with those ECM/Integrin down- or up-regulated genes (Figure 4a), and ITGA6 with modulated chemokine/cytokine genes (Figure 4b). Thus, in each panel, we can visualize again which genes were down- or up-regulated (identified by the green and red names inside the circles, respectively). In respect to the ECM/Integrin networks (Figure 4a), we included the following parameters: co-expression, physical interactions, co-localization, pathways, and genetic interactions. As depicted in the panel, in addition to the well-known physical interactions between integrin subunits and integrin and their corresponding ligands, there were a large number of pathway-related interactions in connection with the knocked-down ITGA6 gene. For chemokine/cytokine network (Figure 4b), we included the co-expression and co-localization parameters. The putative interacting network seen in the panel suggests that effects of ITGA6 knock-down may have a direct effect upon CXCL2 and IL-18, with in turn would modify several other chemokines, through interactions due their co-expression and/or co-localization. Overall, the *in silico *approach used herein indicates that, once ITGA6 gene is knocked-down, several intracellular circuits comprising the expression of membrane and secretory moieties related to cell adhesion and/or cell migration (e.g. integrins, ECM and chemokines) by human TEC may be altered, resulting in multiple changes in cell behavior.

## Discussion

The present work represents the first experimental knock-down of ITGA6 gene in the thymic epithelium, and provides new clues on the complexity of cell migration-related gene networks existing in this tissue.

We first demonstrated that treatment of cultured human TEC with ITGA6 specific siRNA (but not the unrelated scramble counterpart) largely reduced the expression levels of both ITGA6 transcripts and the corresponding CD49f integrin subunit, as determined by qRT-PCR, cytofluorometry and immunofluorescence, respectively. This knock-down affected the functional capacity of TEC to adhere to laminin, the specific ligand of integrin receptors containing α6 subunit, in addition to a loss of the typical stellate profile. By contrast, flow cytometry data strongly indicate that cell viability is not altered. Interestingly, the ITGA6 gene knock-down was paralleled by a significant reduction of the membrane expression levels of other integrin α-chains, such as CD49b, CD49c and CD49d, which form the integrins α2β1 (VLA-2), α3β1 (VLA-3) and α4β1 (VLA-4), receptors for type 1 collagen, laminin and fibronectin, respectively. This is somewhat distinct from the effects we have recently reported in respect to the impairment of CD49e expression (which forms the integrin α5β1/VLA-5, another fibronectin receptor), in which we did not find changes in the membrane expression of VLA-4, ascertained by cytofluorometry as well [7]. Conceptually, these findings support the notion that, although a cross-talk among integrins in the thymic epithelium does occur, it has specificities related to the given integrin whose expression is abrogated.

In any case, these data prompted us to perform a larger cell migration-related gene analyses, now comprising selected 50 genes of ECM ligands and receptors, as well as chemokines. Rather surprisingly, qRT-PCR data revealed that the expression levels of 28 genes out of 50 were significantly changed; 18 being down-regulated and 10 being up-regulated. The expression levels of several transcripts encoding integrin and laminin chains were decreased in ITGA6 siRNA treated TEC. Nevertheless, ITGA3 gene transcription was not significantly altered when comparing ITGA6 siRNA *versus *control siRNA treatment. This is apparently paradoxical with the decrease seen at the membrane expression levels of the corresponding CD49f. Interestingly however, very similar data were recently reported in respect to human keratinocytes: ITGA6 knock-down in these cells also resulted in a decrease of CD49c on the cell membrane but no difference was seen at the transcriptional levels [17], thus suggesting that CD49c expression is translationally regulated following ITGA6 gene knock-down.

It is also interesting to note that we found down- and up-regulation among those genes encoding laminin chains. This finding indicate that some homeostatic circuits may be triggered following ITGA6 gene knock-down, somehow re-shaping the pattern of laminin expression by human TEC.

It is also noteworthy that FN1 gene transcription level by TEC is also down-regulated after ITGA6 gene knock-down, showing that the expression of another ECM ligand/receptor pair (fibronectin/VLA-5) is affected when ITGA6 transcription is impaired.

Lastly, quantitative gene expression analysis also revealed that various chemokine genes are modulated following ITGA6 knock-down, thus unraveling how complex is the control of cell migration-related gene expression in the human thymic epithelium. This scenario is further supported by the *in silico *approach for searching potential interactive networks among the various ECM/integrin and chemokine genes.

## Conclusion

The data presented herein clearly show that down regulation of ITGA6 gene in the human thymic epithelium triggers a complex cascade of effects upon the expression levels of several other cell migration-related genes, including extracellular matrix and chemokine ligands and receptors. In this respect, our findings point to the notion that the expression of several genes involved in the control of thymocyte migration by the thymic microenvironment should be regarded as complex networks, so that a defect in the expression of one single gene may result in an amplified cascade with significant down-regulation of TEC adhesion onto the natural ligand, and potential consequences upon the normal patterns of TEC/thymocyte interactions.

## Methods

### Thymic epithelial cell cultures

The human TEC line was obtained from an infant thymus by an explant technique and limiting dilution cloning [18]. It has been kindly provided by Dr. Maria Luiza Toribio (*Universidad Autonoma de Madrid*, Madrid, Spain). These cells were shown to express constitutively several integrin-type ECM receptors including VLA-4, VLA-5 and VLA-6 laminin [18,19]; being able to interact with thymocytes [19]. Cells were cultured in 10% fetal bovine serum-supplemented RPMI 1640 medium at 37° C in a 5% CO_2 _atmosphere.

### ITGA6 gene silencing

Three ITGA6 specific siRNAs (sc-43129A: 5'- CCAUCACAGUAACUCCUAAtt-3', 5'- UUAGGAGUUACUGUGAUGGtt-3'; sc-43129B: 5'-GGAUAUGCCUCCAGGUUAAtt- 3', 5'-UUAACCUGGAGGCAUAUCCtt-3'; sc-43129C: 5'-CCAAACUGAUCCAGUAUAAtt-3', 5'-UUAUACUGGAUCAGUUUGGtt-3'), and the negative control siRNA (scrambled sequence) were synthesized by Santa Cruz Biotechnology (Santa Cruz Co., CA, USA). Cells were cultured from 60 to 80% confluence in 6-well tissue culture plates and transfected with ITGA6 siRNA (5 nM) with the Lipofectamine-2000 transfection reagent (Invitrogen, CA, USA); control cells were treated with transfection reagent and control siRNA according to the manufacturer's instructions. Cells were incubated 6 hours at 37° C in 5% CO_2 _atmosphere. The transfection mixture was then removed and replaced with normal growth medium. Experiments were conducted 48 hours after transfection.

### RNA isolation, cDNA synthesis and quantitative real-time RT-PCR

Total RNA was isolated from TEC transfected with control scramble siRNA or ITGA6 siRNA using RNeasy Mini Kit (Qiagen, CA, USA), 48 hours after transfection, according to the manufacturer's instructions. Total RNA concentration and purity were determined from the ratio of absorbance readings at 260 and 280 nm using a Nanodrop ND8000 spectrophotometer (Thermo Scientific NanoDrop Products, DE, EUA), and RNA integrity was tested using Agilent 2100 Bioanalyzer (Agilent Technologies, CA, USA). Complementary DNA (cDNA) synthesis reactions were performed using Superscrit II Reverse Transcriptase (Invitrogen) and an StepOnePlus™ Real-Time PCR System (Applied Biosytems, NY, USA) in accordance with the manufacturer's instructions using 2.0 µg of extracted RNA per sample.

Quantitative real-time PCR analysis was performed using olygonucleotides designed to detect expression of selected extracellular matrix ligand and receptors as well as chemokine genes. Fifty genes were analysed (see Additional file 2 Table 1). The PCR primers were designed based on the sequences reported in NCBI GenBank (http://www.ncbi.nlm.nih.gov), using the Primer3 software (http://primer3.wi.mit.edu/). Briefly, the final cDNA products were diluted 10-fold and amplified using FAST SYBR Green Master Mix (Applied Biosystems, NY USA) in a 25 µL reaction mixture that was pipetted into each well of a 96-well optical plate. All standard dilutions were run in triplicate.

Real-time PCR was performed using a two-step cycling program involving an initial single cycle of 95° C for 10 min, followed by 40 cycles of 95° C for 15 s, then 60° C for 1 min in the StepOnePlus™ Real-Time PCR System (Applied Biosystems, NY, USA) with Sequence Detector System software 1.6.3. A first derivative dissociation curve was performed (95° C for 1 min, 65° C for 2 min, then ramped from 65° C to 95° C at a rate of 2° C/min). The formation of a single peak at temperatures higher than 80°C confirmed the presence of a single PCR product in the reaction mixture. The fluorescence accumulation data of real-time RT-PCR duplicate reaction of each sample were used to fit four parameters sigmoid curves to represent each amplification curve using the library qpcR [20] for the R statistical package version 2.14.1 [21]. The cycle of quantification, Cp, was measured at the maximum of the first derivative of the fitted sigmoid curve. The efficiency of each amplification reaction was calculated as the ratio between the fluorescence of the cycle of quantification and fluorescence of the cycle immediately preceding that. Endogenous controls used in the normalization between the different amplified samples were selected among ACTB, B2M, GAPDH, HPRT1 e RPL13A human genes by the method geNorm [22].

### Immunohistochemistry

Transfected TEC were grown in Lab-Tek chambers, washed in PBS and fixed absolute ethanol at room temperature for 10 min. Samples were incubated with BSA 1% to block non-specific sites; being then subjected to the anti-CD49f monoclonal antibody (1:100) (Santa Cruz Co., CA, USA) for 45 min at room temperature. After three gentle PBS washings, the Alexa 546-coupled secondary antibody (Invitrogen, CA, USA) was applied for one hour followed by PBS and nucleus staining with DAPI (Invitrogen, CA, USA). Fluorochrome-labeled Abs was detected using a Zeiss Axio Imager A2 microscope (Carl Zeiss, Oberkochen, Germany). Images were acquired with a CCD camera (Hamamatsu Orca, Shizuoka, Japan). Negative controls, in which primary antibodies were replaced by unrelated immunoglobulin was used alone, and did not generate any significant labeling.

### Flow Cytometry

We also performed cytofluorometric analyses of TEC, for evaluating the membrane density of the following integrin α or β chains: CD49b, CD49c, CD49d, CD49f, CD29 and CD104 (respectively α2, α3, α4, α6, β1 and β4). For that, the cells were immunostained with corresponding fluorochrome labeled antibodies or unrelated Ig isotype-matched controls. All these antibodies were purchased from BD Bioscience (San Jose, CA, USA).

Briefly, cells were washed in PBS, detached using tripsin/EDTA, resuspended in 0.1 ml RPMI-1640 (10^6 ^cells/mL), and treated with fluorochrome-labeled primary antibody or unrelated control for 30 min in a dark chamber. After further PBS washing, the cells were fixed for 15 min using formaldehyde 2%. Acquisition of each fluorescence labeling was performed using a FACSCanto II flow cytometer (BD Biosciences, CA, USA), and analyses were done with the BD FACSDiva 6.1.3 software (BD Biosciences, CA, USA) or the Summit software (Dako, Carpinteria, USA).

In order to evaluate cell viability, in five independent experiments, we analysed the SSC/FSC profiles in both control siRNA and ITGA6-siRNA TEC groups. This procedure provides a differential relative numbers of dying and viable cells, with dying cells bearing very low FSC pattern.

### Cell adhesion assays

Laminina 111 from Engelbreth-Holm-Swarm murine tumor and BSA, both purchased from Sigma-Aldrich, were used to prepare the coating of wells within 24-well tissue culture dishes (Nunc) with 400 µl of solution (10 µg/ml) for 1 hr at room temperature. The blocking of non-specific sites was done with PBS/BSA 1% for 45 min at 37°C in a 5% CO_2 _atmosphere. The transfected cells were centrifuged, re-suspended in 10% fetal bovine serum-supplemented RPMI 1640 medium, and applied to the substrates in a 300 µl drop with 10^4 ^cells at $37^{\underline{{}^{\circ}}}$ C in a 5% CO2 atmosphere. The adhesion assay was stopped by washing off loosely attached cells. The attached cells then were fixed in ethanol for 10 min and stained with Panoptic Solution (LB Laborclin, PR, BR). Adhesion was quantified by light microscopy by counting all attached cells within five microscopic fields in each condition. Within each experiment each condition was performed in triplicates; the results represent the mean ± SE of three independent experiments.

### Statistical analyses

The comparison of means of normalized gene expression values of PCR arrays between the two groups were performed either by a nonparametric one-way ANOVA with 1,000 unrestricted permutations, followed for pair-wise comparisons with Bonferroni adjustment or by a nonparametric *t*-test with 1,000 unrestricted permutations [23] for two or three groups respectively. Results were represented in graphs displaying the expression levels mean ± standard error of mean of each group relative to the control group. Two-tailed levels of significance less than or equal to 0.01, 0.05 and 0.1 were considered as "highly significant" and "significant" and "suggestive", respectively. Also, the relationship between differentially expressed genes and sample profiles was investigated by Bayesian infinite mixtures model bi-cluster analysis [24] and represented by 2D heatmaps and dendograms. Also, networks of potential interactions were inferred using the platform freely available in the framework of the *GeneMANIA database *devised at Toronto University, Canada [25,26].

The statistical analysis of the cytofluorometry and cell adhesion data was performed using Student's *t *test to compare differences between the two groups (unpaired, two tailed), with p-value < 0.05 being considered significant. Data were presented as mean ± standard error of the mean fluorescence intensity.

## List of abbreviations used

ECM: extracellular matrix; FN, fibronectin; LM: laminin; TEC: thymic epithelial cells.

## Competing interests

The authors declare that they have no competing interests.

## Authors' contributions

DCFG performed all the experiments and construction of networks, participated in the interpretation of the results and in the writing of the manuscript. ECS helped in the gene silencing and immunohistochemistry experiments and participated in the interpretation of the results. MRA carried out the statistical analyses and participated in the writing of the manuscript. ATV and WS conceived, designed and coordinated the study, participated in the interpretation of the results and in the writing of the manuscript. All authors read and approved the final manuscript.

## Supplementary Material

Additional file 1 **ITGA6 gene silencing in human thymic epithelial cells does not change the relative numbers of dying cells in culture**. Flow cytometric analysis of TEC shows forward and side scatter parameters of control siRNA (left panel) and ITGA6 siRNA (right panel) transfected cells. The numbers inside the plot area indicate the percentages ± SD of cells inside the rectangles, that correspond to dying and living cells (smaller and larger rectangles, respectively). Mean values were obtained from 5 independent experiments. Click here for file

Additional file 2 Table 1: List of studied genes Click here for file

Additional file 3 Table 2: Specificity of anti-ITGA6 nucleotide sequences Click here for file

## Declarations

Publication for this article has been funded by CNPq (Conselho Nacional de Desenvolvimento Científico e Tecnológico), Capes (Coordenação de aperfeiçoamento de pessoal de nível superior), Faperj (Fundação de Amparo à Pesquisa do Estado do Rio de Janeiro) and Oswaldo Cruz Foundation (Brazil).

This article has been published as part of *BMC Genomics *Volume 14 Supplement 6, 2013: Proceedings of the International Conference of the Brazilian Association for Bioinformatics and Computational Biology (X-meeting 2012). The full contents of the supplement are available online at http://www.biomedcentral.com/bmcgenomics/supplements/14/S6.
