# Supplementary figures and images for: ITGA6 gene silencing by RNA interference modulates the expression of a large number of cell migration-related genes in human thymic epithelial cells
Source: BMC Genomics. 2013 Oct 25;14(Suppl 6):S3. doi: 10.1186/1471-2164-14-S6-S3 (PMC3909006; doi:10.1186/1471-2164-14-S6-S3)

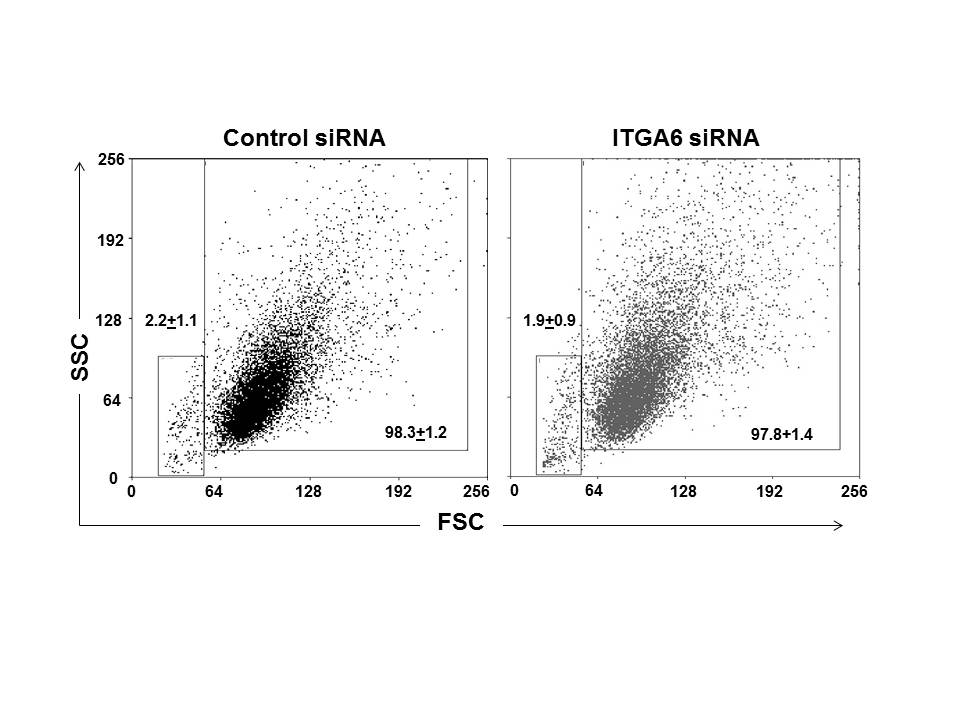

Supplement: Additional file 1 — ITGA6 gene silencing in human thymic epithelial cells does not change the relative numbers of dying cells in culture. Flow cytometric analysis of TEC shows forward and side scatter parameters of control siRNA (left panel) and ITGA6 siRNA (right panel) transfected cells. The numbers inside the plot area indicate the percentages ± SD of cells inside the rectangles, that correspond to dying and living cells (smaller and larger rectangles, respectively). Mean values were obtained from 5 independent experiments. [file 1471-2164-14-S6-S3-S1.jpg]
