# Supplementary material for: ITGA6 gene silencing by RNA interference modulates the expression of a large number of cell migration-related genes in human thymic epithelial cells
Source: BMC Genomics. 2013 Oct 25;14(Suppl 6):S3. doi: 10.1186/1471-2164-14-S6-S3 (PMC3909006; doi:10.1186/1471-2164-14-S6-S3)
Supplement: Additional file 2 — Table 1: List of studied genes [file 1471-2164-14-S6-S3-S2.PDF]

List of genes used for the quantitative RT-PCR arrays

Group	Gene Symbol	GenBank	Corresponding polypeptide
Integrin subunits	ITGA1	NM_181501.1	Integrin, α 1 chain
	ITGA2	NM_002203	Integrin, α 2 chain (CD49b, α 2 subunit of VLA-2 receptor)
	ITGA3	NM_002204.2	Integrin, α 3 chain (CD49c, α 3 subunit of VLA-3 receptor)
	ITGA4	NM_000885	Integrin, α 4 chain (CD49d, α 4 subunit of VLA-4 receptor)
	ITGA5	NM_002205.2	Integrin, α 5 chain (CD49e, α5 subunit of VLA-5 receptor)
	ITGA6	NM_000210.2	Integrin, α 6 chain (CD49f, α6 subunit of VLA-6 and α 6 β 4 receptor)
	ITGA7	NM_002206.2	Integrin, α 7 chain (laminin receptor)
	ITGA8	NM_003638.1	Integrin, α 8 chain
	ITGAL	NM_002209.2	Integrin, α L chain (CD11A, lymphocyte function-associated antigen 1)
	ITGAV	NM_002210.3	Integrin, α V chain (CD51, vitronectin receptor)
	ITGB1	NM_002211.3	Integrin, β 1 chain (CD29, beta polypeptide)
	ITGB2	NM_000211.3	Integrin, β 2 chain
	ITGB3	NM_000212.2	Integrin, β 3 chain (CD61, platelet glycoprotein IIIa)
	ITGB4	NM_000213.3	Integrin, β 4 chain (CD104, beta polypeptide)
	ITGB5	NM_002213.3	Integrin, β 5 chain
Laminin isoforms and Fibronectin	LAMA1	NM_005559.3	Laminin, α 1 chain
	LAMA2	NM_000426	Laminin, α 2 chain
	LAMA3	NM_000227.3	Laminin, α 3 chain
	LAMB1	NM_002291	Laminin, β 1 chain
	LAMC1	NM_002293.3	Laminin, γ 1 chain
	FN1	NM_002026	Fibronectin 1
Chemokines	CCL2	NM_002982	Chemokine (C-C motif) ligand 2
	CCL3	NM_002983.2	Chemokine (C-C motif) ligand 3
	CCL4	NM_002984	Chemokine (C-C motif) ligand 4
	CCL5	NM_002985.2	Chemokine (C-C motif) ligand 5
	CCL7	NM_006273	Chemokine (C-C motif) ligand 7
	CCL8	NM_005623	Chemokine (C-C motif) ligand 8
	CCL11	NM_002986	Chemokine (C-C motif) ligand 11
	CCL13	NM_005408	Chemokine (C-C motif) ligand 13
	CCL16	NM_004590	Chemokine (C-C motif) ligand 16
	CCL17	NM_002987.2	Chemokine (C-C motif) ligand 17
	CCL18	NM_002988	Chemokine (C-C motif) ligand 18
	CCL19	NM_006274.2	Chemokine (C-C motif) ligand 19
	CXCL1	NM_001511	Chemokine (C-X-C motif) ligand 1
	CXCL2	NM_002089	Chemokine (C-X-C motif) ligand 2
	CXCL3	NM_002090	Chemokine (C-X-C motif) ligand 3
	CXCL5	NM_002994	Chemokine (C-X-C motif) ligand 5
	CXCL6	NM_002993	Chemokine (C-X-C motif) ligand 6
	CXCL9	NM_002416	Chemokine (C-X-C motif) ligand 9
	CXCL10	NM_001565	Chemokine (C-X-C motif) ligand 10
	CXCL11	NM_005409	Chemokine (C-X-C motif) ligand 11
	CXCL12	NM_000609	Chemokine (C-X-C motif) ligand 12
	CXCL13	NM_006419	Chemokine (C-X-C motif) ligand 13
	CX3CL1	NM_002996	Chemokine (C-X3-C motif) ligand 1
Cytokines	IL1A	NM_000575.3	interleukin-1 α
	IL4	NM_000589	Interleukin-4
	IL8	NM_000584	Interleukin-8
	IL13	NM_002188.2	Interleukin-13
	IL16	NM_004513	Interleukin-16
	IL18	NM_003855.2	Interleukin-18
