# Supplementary material for: ITGA6 gene silencing by RNA interference modulates the expression of a large number of cell migration-related genes in human thymic epithelial cells
Source: BMC Genomics. 2013 Oct 25;14(Suppl 6):S3. doi: 10.1186/1471-2164-14-S6-S3 (PMC3909006; doi:10.1186/1471-2164-14-S6-S3)
Supplement: Additional file 3 — Table 2: Specificity of anti-ITGA6 nucleotide sequences [file 1471-2164-14-S6-S3-S3.PDF]

Specificity of anti-ITGA6 nucleotide sequences, as revealed by the BLAST software (see ref. 27)

SubjectID	QueryID	Perc.Ident	Align.Length	Mismatches	Gap.Openings	mRNA biding site	E-value	Bits
siRNA 1 sense (sc-43129A)	Integrin alpha 6 (ITGA6), transcripts 1 and 2, mRNA	100%	19	0	0	1621 to 1639	0.088	38.2 (19)
	ST6 (alpha-N-acetyl-neuraminyl-2,3-beta-galactosyl-1,3)-N-acetylgalactosaminide alpha-2,6-sialyltransferase 3 (ST6GALNAC3), transcript variant 2, mRNA	100%	15	0	0	742 to 756	21	30.2 (15)
siRNA 1 anti-sense (sc-43129B)	Integrin alpha 6 (ITGA6), transcripts 1 and 2, mRNA	100%	21	0	0	1619 to 1639	0.006	42.1 (21)
	ST6 (alpha-N-acetyl-neuraminyl-2,3-beta-galactosyl-1,3)-N-acetylgalactosaminide alpha-2,6-sialyltransferase 3 (ST6GALNAC3), transcript variant 2, mRNA	100%	15	0	0	742 to 756	21	30.2 (15)
siRNA 2 sense (sc-43129B)	Integrin alpha 6 (ITGA6), transcripts 1 and 2, mRNA	100%	19	0	0	1684 to 1702	0.088	38.2 (19)
siRNA 2 anti-sense (sc-43129B)	Integrin alpha 6 (ITGA6), transcripts 1 and 2, mRNA	100%	19	0	0	1685 to 1702	0.088	38.2 (19)
siRNA 3 sense (sc-43129C)	Integrin alpha 6 (ITGA6), transcripts 1 and 2, mRNA	100%	19	0	0	4715 to 4733	0.088	38.2 (19)
siRNA 3 anti-sense (sc-43129C)	Integrin alpha 6 (ITGA6), transcripts 1 and 2, mRNA	100%	19	0	0	4715 to 4733	0.088	38.2 (19)
